# Nitrite of Amyl and Belladonna in Dysmenorrhœa

**Published:** 1875-01-15

**Authors:** 


### NITRITE OF AMYL AND BELLADONNA IN DYSMENORRHŒA.

New York Medical Journal and Library Association,

Stated Meeting, Nov. 27, 1874.

THE paper for the evening was read by Dr. Mary Putnam Jacobi, upon "Nitrite of Amyl and Belladonna in Dysmenorrhœa."

The clinical history of three cases was given to illustrate the method of operation of the above remedies. All three were cases of severe spasmodic dysmenorrhœa. They were treated by administering belladonna for several days previous to the recurrence of menstruation, and nitrite of amyl by inhalation during the paroxysm. This treatment, it was believed, had a rational basis.

The argument in its support was founded upon the data furnished in the second case, in which were manifest three sets of phenomena:

(1.) Vomiting, pallor of skin, cold hands and feet.

(2.) Extraordinary peristaltic action of the intestines.

(3.) Spasmodic pain in the uterus.

All these point toward one element—namely, that of spasmodic contraction of blood-vessels.

First, the so-called sympathy between the uterus and the stomach, and between the stomach and brain, were fully considered in their dependence and interdependence with reference to the symptom, *vomiting*.

It was believed, reasoning from the experiments of Schiff and others, that the vomiting of pregnancy, vomiting of sea-sickness, and many cases analogous in character, was due to the same cause, namely, anæmia of the brain, producing spasmodic contraction of blood-vessels at its base.

It was farther argued that anæmia of the intestines produces contractions or increased peristalsis, due to spasmodic contraction of blood-vessels.

There are three conditions in which a hollow muscular organ can contract in the state of vacuity:

(1.) After direct irritation of its nerves.

(2.) After direct irritation of its muscular fibre.

(3.) After changes in its circulation.

A detailed account of six experiments was given. The experiments had been performed upon rabbits. The abdominal cavity was opened, the intestines drawn out and carefully protected in a bag of oil-silk, which was kept immersed in a vessel of warm water; the uterus exposed, and the abdominal aorta exposed. The aorta was then compressed with a ligature, and the result carefully noted.

Several waves of peristalsis ran *down* the rectum, but never in a contrary direction. Contraction of the uterus occurred, and was distinctly visible at the middle third of the organ. Upon removal of the ligature the contractions ceased. The time at which contractions appeared after compression of the aorta was made, also the duration of the contraction after compression had been removed, were carefully noted. The conclusion made from the experiments was, that tonic contractions of the uterus may be excited by occlusion of the aorta, and that such contractions continue from one to four minutes after compression has been removed. Clonic contractions also occurred, after the type of contractions of masses of smooth muscular fibre.

What bearing do the results of these experiments have upon the treatment of spasmodic dysmenorrhœa?

The pain in these cases is dependent upon tonic and clonic contraction of the uterus.

These, in turn, are dependent upon some cause. Of the conditions in which a hollow muscular organ can contract in a state of vacuity, direct irritation of muscular fibre and direct irritation of nerves were excluded. Consequently we are obliged to fall back upon changes in the circulation of the uterine walls. If the change of the blood-vessels passes to an irritation, spasmodic contraction must take place, and uterine contractions will be determined by local anæmia.

Spasmodic contraction of blood-vessels resulting from irritation of vaso-motor nerves, is the cause of the pain of spasmodic dysmenorrhœa. It is upon these considerations that the remedies suggested are used. The *secondary* effect of belladonna is dilatation of the blood-vessels.

Belladonna is to be administered, therefore, previous to the occurrence of menstruation, for the reason that it is desirable to obtain the *secondary* effects of the remedy,

Nitrite of amyl is used for the purpose of relaxing blood-vessels. This is in accordance with the admitted physiological action of the remedy.

This method of treatment, of course, is more especially adapted to cases of spasmodic dysmenorrhœa; but it has been found, both in the experience of the author of the paper, and in that of others, that great relief may be afforded even in those cases in which the dysmenorrhœa depended upon displacements, constriction of the cervix, etc.

The method is, to administer belladonna in ordinary doses for several days previous to the occurrence of the menstrual flow, and when pain comes, to administer by inhalation from two to six drops of the nitrite of amyl, p. r. n. In one case a single drop of amyl was all that was required.

Dr. Sell remarked that he had been in the habit of administering nitrite of amyl by the mouth, and had obtained just as good results as he had obtained when the remedy had been inhaled. He prescribed it in one-drop doses, combined with drachm doses of peppermint water, and repeated every half hour. In one case of dysmenorrhœa, and one only, he had used the nitrite of amyl, and in that case the patient was completely relieved of pain.

Drs. Pallen and Peaslee did not feel prepared to discuss the paper.—*N. Y. Medical Record.*

"Upon the Researches of Curry, and the Recent Views with Regard to the remedial Use of Water."—The researches of Curry were made at the close of the last and the beginning of the present century. It appeared from the reading of the paper, that Curry resorted to the use of water extensively in the treatment of a great variety of diseases. Not only were his observations made with regard to the effect of cold, but also of warm water. The results of his experience were extensively quoted, and the conclusion was easily drawn, that the water treatment of to-day is essentially a revival of a practice adopted three-fourths of a century ago. It was an interesting fact, also, that Curry appreciated and clearly set forth the importance of the thermometer, and indeed the present axillary and self-registering thermometer were anticipated by him. The quotations from Curry, although so old, sounded like the fresh observations of a master-mind upon this subject—so fully were they in keeping with the most recent views concerning the value of this remedial agent.

Prof. Flint's method of using the Cold Pack is as follows:

Wrap the body in a sheet wet in cold water, and then sprinkle with a watering-pot, and continue the pack from ten minutes to half an hour, according to the temperature and condition of the pulse. Used in this manner, he is of the opinion that we obtain all the benefits of the *bath.*

Special reference was made to the internal use of water in the treatment of disease. The quantity to be taken in the treatment of fever should be regulated by the feelings of the patient. Air, food, and drink: these form the tripod whence emanate the laws which should govern the hygienic management of our cases of fever. The internal use of water was suggested in the treatment of renal affections. A case was related in which the urine was scanty, albuminous, and of very low specific gravity, accompanied by other symptoms of grave character, but was brought to a successful termination by water treatment, originated and executed by Dr. Perry, formerly house physician at Bellevue Hospital. The chief feature of the treatment was the administration of four ounces of water, or milk and water every half hour, regularly and steadily persisted in.—Dr. Austin Flint, in paper read before N. Y. Academy of Medicine.—*N. Y. Med. Record.*

---

Ergotin in Croupous Pneumonia.—Dr. Wycisk, acting on the principle that this drug contracts the vessels, and so prevents exudation from them, has treated six cases of croupous pneumonia in this way, and gives the following report: In one such case, marked by an excessive highly albuminous expectoration, it ceased entirely two hours after the administration of the drug in powders of nine grains each every quarter of an hour. The abundant rales in both lungs soon diminished, so that there was only a slight crepitation at the original focus of the disease. These good effects lasted for two days. Two relapses occurred, however, but the same good effect was again obtained by the administration of the ergot. After the second relapse, ten drop doses of the tincture of ergot were given four times daily, until convalescence was fully established. In the five other cases the ergot was used early, and none ended fatally; none became chronic; and none left appreciable deposits behind them; in all of them the exudation was decidedly checked by the ergot. It is, however, held to be a dangerous remedy when the lungs are considerably infiltrated, where there is emphysema, where the cerebral arteries are weak, and where the patients are feeble or decrepid.—*Allg. Med. Central Ztg.*, 88, 1874.—*N. Y. Med. Record.*

---

Dr. E. Warren Sawyer, a graduate of the Harvard Medical School, has obtained the position of Lecturer on Obstetrics at the Rush Medical College, after a competitive examination with thirteen others.—*N. Y. Med. Record.*

MEDICAL GRADUATES.—The Medical Colleges of the United States graduated in the year 1874, three thousand students. — *N. Y. Medical Record.*

PUERPERAL FEVER IN THE NEW MATERNITY AT BONN.—In a letter to *The Lancet* Nov. 28, 1874, a correspondent writes, concerning the new Gynæcological Institute at Bonn:

"It is a singular fact that puerperal fever broke out immediately after the opening of the hospital, and six patients died from it, while since that time there has not been a single case of the disease."—*N. Y. Med. Record.*

A CANINE SURGEON.—An interesting instance of sagacity has been recorded by a correspondent of the *Philadelphia Medical Times.* A large dog of the St. Bernard and Newfoundland breed, called "Carlo," had been brought up with a cat, and the two became great friends. The cat had the misfortune to swallow a needle and thread which had got mixed up with some minced-meat prepared for its food. The cat suffered intensely. The dog, after some apparent deliberation, fixed upon a spot in the cat's neck, and commenced to lick it, which operation it continued to perform very assiduously for the better part of two days, his feline friend aiding him by keeping its head on one side. All at once the dog got much excited, and was seen to be anxiously striving to catch something between its teeth. The animal succeeded at last, by a sudden jerk, in seizing the point of the needle, and drawing it through the skin and fur; but it still hung by the thread, which was divided by the daughter of the dog's master. The dog subsequently, after greeting the return of its master's son, immediately went to the place where the needle and thread had been thrown. Dr. Benjamin Lee, who relates the circumstance, says that he has been at the pains to verify the facts, and to do himself the pleasure of shaking "Carlo's" paw, and giving him the salutation of a *confrere.*—*London Lancet.*

THE BRITISH MEDICAL ASSSOCIATION will meet in Edinburgh next year. Preparations have been begun, and "lively and pleasant expectations" of the visit are already indulged in.

A CURE FOR BRIGHT'S DISEASE.—Dr. Hegswald says, a half pint thrice daily, of a fresh infusion of the leaves of *Asplenium Scolopendrium*, L., is a most successful treatment in Bright's disease. This is the harts-tongue or spleenwort, and is said to be popular in Devonshire and elsewhere, for its medicinal virtues.—*Phil. Med. and Surg. Rep.*

DANGER FROM EXCAVATIONS.--It is well known that the exposure of large quantities of fresh earth, as attends railroad and canal construction, develops intermittent and typho-malarial fevers. To lessen this, Dr. Stephen Smith, of New York City, offers the wise recommendation that sewers be laid only after November 15th, and before June 1st; and last Tuesday week the Board of Health, accordingly, resolved that the Commissioner of Public Works be requested to omit all excavations between June 15th, and October 1st, and that no subsoil be exposed during that time.—*Phil. Med. and Surg. Rep.*

TUBERCULOSIS NOT INOCULABLE.—In a late communication to the Academy of Sciences of Paris, M. Metzquer tried to upset Villemin's doctrine. For the last five years the author has made experiments (from seventy to eighty) under the direction of Prof. Feltz, of the Faculty of Nancy. He never succeeded in inducing pulmonary consumption in the inoculated animals. The results were capillary embolism, infarctus, vesicular pneumonia, &c., all of which lesions have (the author maintains) been confounded with tubercle. Tuberculosis may, however, be generated in animals (says M. Metzquer), without inoculation of tubercular matter, by rough treatment, bad food, and, strange to say, by inflicting a wound upon them.—*London Lancet.*

SUPPRESSION OF URINE FOR TWENTY-FIVE DAYS.— Dr. A. W. Fontaine (*Virginia Medical Monthly*) reports a case in which the above phenomenon took place. Patient was a lady, aged twenty-one, nervo-sanguine temperament. The urinary suppression took place during an attack of intermittent fever. Diuretics one and all failed utterly to restore the action of the kidneys. Cupping, blisters and galvanism were alike futile. On the twenty-fourth day an injection into the bladder was made with twenty drops tinct. cantharides in a half pint of warm water. The next day a little urine was seen, which, under the same treatment, gradually increased to normal amount. Other cases carefully observed, are noted, in which suppression of urine continued respectively eleven days, sixteen weeks, twenty-two weeks, fifteen months, and lastly, a youth, seventeen years old, who never made water in his life, and was perfectly vigorous and active. — *Detroit Medical Journal.*

---

LETZERICH ON LOCAL AND GENERAL DIPHTHERIA.—Letzerich has recently published his views on this subject, which have special interest, as he is one of the boldest defenders of the parasitic theory of diphtheria, and has contributed some very important data for the support of his views. He states his belief as follows: Local diphtheria is a contagious disease of the mucous membrane. The *contagium* consists of a fungous vegetation which goes through its development and growth in the diphtheritic exudation, and in the tissue of the mucous membrane if there is no exudation. Should these low organisms force their way into the circulation, they spread and increase in the body, and work a general infection; *i. e.*, general diphtheria.

These low organisms which he classes as bacteria, plasma spheres and micrococci, etc., induce various changes both primarily in the local foci of the disease; and secondarily, in such organs as the kidneys, spleen, liver and heart. First come disturbances in the nutrition of the tissues themselves, from the occurrence of fungous emboli; these interfere with the circulation, and then an astonishing increase in the fungi takes place. With the increase of the micrococci is *pari passu*, a destruction of the cellular elements, so that the cells of the kidneys, liver, and spleen, may disappear, as well as the contractile substance of the cardiac muscle.—*Virchow's Archiv.*, 4, 1874.—*N. Y. Med. Record.*

---

MR. ERICHSEN ON AMERICAN SURGERY.—On November 9th, Mr. Erichsen delivered an address in London, on his impressions of American surgery during his late tour in this country. It is in general favorable, as may be judged from the following extract:—

Surgery in the United States certainly stands at a very high level of excellence. The hospital surgeons throughout the country have struck me as being alike practical, progressive, and learned in a very high degree. In practical skill, and aptitude for mechanical appliances of all kinds, they are certainly excelled by no class of practitioners in any country. They are thoroughly up to modern surgery in its most progressive forms, and I have never met with any class of men who are so well read and so perfectly acquainted with all that is done in their profession outside their own country. It would be a great injustice to American surgeons for it to be supposed that surgical skill is confined to the large cities, or to the few. On the contrary, I know no country in which, so far as it is possible to judge from the contemporary medical literature, there is so widely diffused a high standard of operative skill, as in the country districts and more remote provinces of the United States. The bent of the mind of the American surgeon is, like ours, practical rather then scientific; in fact, there are the same mental characteristics displayed in him that we find here; the same self-reliance, the same practical aptitude, the same *curative* instinct, which leads him to consider his patient rather as a human being to be rescued from the effects of disease or injury, than as a scientific object to be studied for the advance of professional knowledge. How, indeed, can it be otherwise than that there should be such a resemblance ?—*Phil. Med and Surg. Rep.*

